# A comparison of machine learning methods for quantifying self-grooming behavior in mice

**DOI:** 10.3389/fnbeh.2024.1340357

**Published:** 2024-01-29

**Authors:** Kassi Correia, Raegan Walker, Christopher Pittenger, Christopher Fields

**Affiliations:** ^1^Department of Psychiatry, Yale School of Medicine, Yale University, New Haven, CT, United States; ^2^Department of Psychiatry, Yale School of Medicine, New Haven, CT, United States

**Keywords:** behavior, HomeCageScan, ARB, machine learning, grooming

## Abstract

**Background:**

As machine learning technology continues to advance and the need for standardized behavioral quantification grows, commercial and open-source automated behavioral analysis tools are gaining prominence in behavioral neuroscience. We present a comparative analysis of three behavioral analysis pipelines—DeepLabCut (DLC) and Simple Behavioral Analysis (SimBA), HomeCageScan (HCS), and manual scoring—in measuring repetitive self-grooming among mice.

**Methods:**

Grooming behavior of mice was recorded at baseline and after water spray or restraint treatments. Videos were processed and analyzed in parallel using 3 methods (DLC/SimBA, HCS, and manual scoring), quantifying both total number of grooming bouts and total grooming duration.

**Results:**

Both treatment conditions (water spray and restraint) resulted in significant elevation in both total grooming duration and number of grooming bouts. HCS measures of grooming duration were significantly elevated relative to those derived from manual scoring: specifically, HCS tended to overestimate duration at low levels of grooming. DLC/SimBA duration measurements were not significantly different than those derived from manual scoring. However, both SimBA and HCS measures of the number of grooming bouts were significantly different than those derived from manual scoring; the magnitude and direction of the difference depended on treatment condition.

**Conclusion:**

DLC/SimBA provides a high-throughput pipeline for quantifying grooming duration that correlates well with manual scoring. However, grooming bout data derived from both DLC/SimBA and HCS did not reliably estimate measures obtained via manual scoring.

## Background

Behavioral studies of laboratory animal models are essential to neuroscience. Although behavioral data is relatively easily acquired, standardizing behavioral analyses across different laboratories poses a challenge (Sousa et al., 2006). Human observation with manual scoring of target behaviors is commonly used, but this method is time and labor intensive, unstandardized, and prone to inter-rater variability (von Ziegler et al., 2021). Observational quantification of behavior is particularly difficult for behaviors that occur rarely, that occur over a long period of time, or that are not readily distilled into a simple parameter such as velocity, time, or frequency of occurrence (Spink et al., 2001).

One solution to these problems is automated analysis from video. Developments in machine learning—the process by which algorithms learn from data—have enabled high-throughput programs capable of handling large amounts of data (Deo, 2015; Yin et al., 2017). When compared to manual analysis by human experimenters, automated behavioral analysis has multiple advantages, including efficiency, standardization, and scalability. In response to the growing demand for high-validity, high-throughput automated behavioral analysis algorithms, the number of commercial and open-source machine learning programs dedicated to behavioral analysis continues to increase (Maekawa et al., 2020; Vonstad et al., 2020; Wiltschko et al., 2020; Wotton et al., 2020; Hsu and Yttri, 2021; Lorsch et al., 2021; Luxem et al., 2022). These tools can be broadly divided into three classes: those for pose estimation, those for complex behavioral analysis, and those for both purposes. This paper will address one solution in each class: DeepLabCut (DLC), Simple Behavioral Analysis (SimBA), and HomeCageScan (HCS; CleverSys, Reston, VA).

Markerless pose estimation tools apply computer vision and machine learning algorithms to automatically detect key body parts across video frames rather than manually attaching visible tags that may fall off or influence natural behavior (Mathis et al., 2018; Vogt, 2021). For example, DeepLabCut is trained by the user to build a model that can consistently recognize the nose, ears, limbs, etc. in frames from new videos by modeling their visual features such as shape, orientation, texture. The trained network then labels new footage by locating these learned body components in each frame, linking them together into pose skeletons depicting the animal’s movements and actions. To elaborate, DeepLabCut utilizes deep neural networks, structured in layered nodes mimicking biological brains, which requires relatively few training examples (~50–200 frames) to achieve human-equivalent accuracy in labeling novel inputs (Mathis et al., 2018; Nath et al., 2019). DLC’s networks are trained by the user, teaching the system to accurately recognize target body parts from labeled frames. After the experimenter has trained the network, it can be reused to label novel behavioral data collected using the same experimental setup. Additionally, once customized on experiment videos, the network can reliably label new footage enabling efficient annotation supporting high-throughput analysis. DLC’s accessibility, including a graphical-user interface (GUI), Jupyter Notebook guidance, and Google Collab graphical processing unit (GPU), have all contributed to its popularity.

Following pose estimation with software such as DeepLabCut, programs like Simple Behavioral Analysis (SimBA) can use pose data to assess and label sequences of positional data as user-defined behavioral phenotypes. SimBA creates predictive classifiers of rodent behavior with millisecond resolution and accuracy that claims to outperform human observation. SimBA supports a four-step pipeline: (1) pre-processing videos, (2) managing pose-estimation data, (3) creating, performing, and analyzing behavioral classifications, and (4) visualizing results (Nilsson et al., 2020).

DLC and SimBA perform pose estimation and behavioral analysis separately. Commercial software packages such as HomeCageScan (HCS; CleverSys, Reston, VA) are dual-purpose, conducting both pose estimation and behavioral analysis. HCS developers state that it can quantify over 38 mouse behaviors, and that the program has been validated in quantifying behavior in healthy mice and in mice with neurodegenerative diseases (Steele et al., 2007).

Despite the advantages of automated analysis, no consensus has been reached as to which protocols should be followed, or on the comparative validity of these programs. Given the recent popularization of supervised machine learning tools and the sheer number of options available, assessing the efficacy of different programs is crucial.

One relevant application of machine-learning informed behavioral analysis is in the study of abnormal repetitive behaviors (ARBs); these are behaviors that are inappropriate, repetitive, and unvarying in goal or motor pattern (Garner, 2005). ARBs can occur and fluctuate over long time periods and are difficult to reduce to simple parameters, problematizing observational scoring (Spink et al., 2001). ARBs have been associated with an array of human psychiatric disorders such as autism spectrum disorder (ASD), Tourette syndrome, obsessive compulsive disorder (OCD), Parkinson’s disease, and others, and are often reported in animal models that seek to capture the pathophysiology of these conditions (Silverman et al., 2010; Muehlmann and Lewis, 2012; Gruner and Pittenger, 2017). The nature of ARBs—their frequency and variability—as well as their potential translational significance underscores the need for robust behavioral analysis, and makes them a target for testing automated, high-throughput strategies for quantification.

In mouse models, one prominent abnormal repetitive behavior is pathological self-grooming, a phenomenon that can be contrasted with the normal grooming behaviors extensively documented in rats by Bolles (1960) and shown to be an innate, repetitive behavior which does not require sensory input in mice by Fentress (1973). Self-grooming encompasses a complex, sequential syntax critically involving corticostriatal control with additional integration across corticolimbic structures (Berridge, 1989; Spruijt et al., 1992; Kalueff et al., 2016). As a stereotypic behavior with intuitive comparisons across species, translational psychiatry increasingly recognizes pathological grooming alterations in mice as highly valuable proxies to model compulsive sequential psychopathology underlying disorders like autism and OCD (Garner and Mason, 2002; American Psychiatric Association, 2013; Bortolato and Pittenger, 2017).

Measuring grooming duration helps to clearly identify genetic and pharmacological factors that affect the regulation of repetitive behaviors. Analyzing this complex repetitive behavior requires precise, high-throughput analytical tools (Kalueff et al., 2007; Ahmari et al., 2013)—a key motivation behind this work. Moreover, because grooming manifests unpredictably across prolonged periods, developing accurate techniques to measure overall expression enables revealing pathogenesis while supporting therapeutic development (Sachs, 1988; Kalueff et al., 2016; Song et al., 2016).

We focus on mice here due to their broad appeal to preclinical researchers. Mice confer advantages in established genetic manipulation lines compared to rats. Their smaller size also facilitates higher density housing that is critical for large interventions. Additionally, excessive grooming in mice provides a well-validated approach to model repetitive behavioral pathologies relevant to psychiatric disease research, our primary focus.

Here, we quantify whole mouse grooming episodes using two automated behavioral analysis pipelines: DeepLabCut/SimBA and HomeCageScan. The output of each program is compared to the other and to manual scoring. We studied self-grooming in C57BL/6 mice across three conditions: at baseline, after restraint stress to evoke spontaneous excessive grooming, and after water spray to provoke exaggerated grooming. Both restraint (van Erp et al., 1994) and water spray protocols (Smolinsky et al., 2009) are well-established methods to induce excessive self-grooming in mice. By parsing grooming into these categories, we can systematically contrast automated quantification during natural conditions versus following two distinct induction procedures known to elevate this repetitive behavior in different manners. We discuss current obstacles in machine learning behavioral analysis and suggest directions for future work.

## Methods

### Animals

All procedures were approved and overseen by the Yale University Institutional Animal Care and use Committee (IACUC). 12 weeks-old male and female C57BL/6 mice weighing 20–30 g were obtained from Jackson Labs (Bar Harbor, ME). Animals were housed in a facility at a temperature of 24°C (297.15 K), relative humidity of 30%–50%, and controlled lighting with light on from 7:00 to 19:00 daily.

### Experimental procedure

Mice, comprising 12 males and 12 females, were pseudo-randomly assigned to two treatment conditions: restraint stress and water spray. This assignment was conducted in a balanced and alternating order, determined by a computer-generated random sequence, to ensure equal distribution across both conditions. Prior to each treatment (collectively referred to as “induction”), baseline behavior was recorded for 10 min. Immediately following induction, behavior was again recorded for 10 min. Recordings were conducted in the HCS apparatus (CleverSys, Reston, VA). After each trial, the behavior box was cleaned with 70% ethanol applied using paper towels, allowed to fully dry, and feces removed if necessary, before introducing the next subject. Videos were processed through the HCS pipeline and, in parallel, exported for analysis in DLC/SimBA. Seven days later, mice were assigned to the other treatment condition (water spray or restraint stress), and additional 10 min baseline and induction measurements were obtained. This resulted in four 10 min observations per mouse.

### HomeCageScan video acquisition

Mice were housed together (4 per cage) and first habituated for 20 min in the behavior room prior to individual recording trials. For video capture, subjects were placed separately into clear plexiglass cages (47 cm length × 36.8 cm width × 20.3 cm height) inside one of four cabinet stations of the Home Cage Rack system (CleverSys Inc., Reston, VA).

Each station contains a camera and lighting fixed at a side angle view. The Pacific PA-290 analog cameras connect to a quad video multiplexer consolidating the 4 feeds into one NTSC signal. This interfaces with a WinTV PCI/USB card (models 350/1950/1955) in a PC capturing uncompressed 30 fps video at 720 × 480 resolution. Onboard multiplexing splits this into four 360 × 240 resolution 30 fps MPG videos per each mouse cage.

### Baseline measurements

Baseline behavior was recorded for 10 min on each of the two sessions, beginning after the last mouse was placed in its cage. Individually recorded videos of each of the four mice are stitched together by the CleverSys system into a single 10 min video containing the behavior of all four animals.

### Water spray treatment protocol

In the water-spray condition, following 10 min of baseline recording, a new recording was started. Each mouse was removed from its cage, placed on a workbench counter and secured at the tail base, and sprayed 30 times on the back with distilled water from a spray bottle held 5–10 cm. away. The mouse was then returned to its cage, and the video timestamp at which the mouse was returned was documented. Recording continued until 10 min after the last mouse had been returned to the HCS cage.

### Restraint-stress treatment protocol

In the restraint stress condition, following 10 min of baseline recording, each mouse was removed from its cage and placed head-first into a ~4 cm diameter-plastic tube, with a breathing hole at one end. Once the animal was entirely in the tube, the other end of the tube was capped, and the tube was left on the workbench for 10 min. After 10 min of restraint each mouse was removed from its tube, immediately returned to its HCS cage, and recorded for 10 min.

### Video preparation

For downstream analysis, we converted MPG files into MP4 format using VLC Media Player’s “Keep Original Video Track” setting, retaining original properties while allowing broader software compatibility. The resulting same-resolution 30 fps MP4 files were utilized across manual scoring, DeepLabCut pose labeling, and SimBA classification methods.

Each resulting MP4 video was uploaded to spark.adobe.com and cropped into 4 separate videos containing one mouse per video and trimmed to the ten minutes of interest (either beginning at the start of the baseline trials, or beginning immediately after the animal was returned to the recording box following restraint stress of water-spray treatment). This resulted in a total of 96 .mp4 videos (24 mice recorded across two baselines and two treatments, water spray and restraint), maintaining the 30 fps frame rate of the original video.

### Technical documentation

DeepLabCut (DLC) and SimBA were implemented remotely at the Yale Center for Research Computing, Atop of a Windows OS. Python v. 3.6.13 was run using 7 32G GPUs. The GUIs of DLC and SimBA were implemented with XQuartz 11 and Tk v. 8.6.10.

### DeepLabCut

The 96 pre-processed 30 fps MP4 videos showing individual mice across conditions served as inputs to the DeepLabCut (DLC, v.2.2.0) pipeline for labeling anatomical body parts in each frame. We defined 8 landmark tags for body tracking: nose, left/right ears, front/back paws, and mid-back. An experimenter exhaustively hand-labeled all instances of these parts across 380 diverse frames selected from 19 videos. This initial tagged dataset forms the foundation for DeepLabCut’s machine learning training process.

In DeepLabCut, we selected ResNet-50 as the foundation for constructing our machine learning model. This ResNet (residual network) architecture developed by Microsoft researchers is optimized for image recognition (He et al., 2016). The name refers to shortcut connections that let information bypass layers, with 50 designating 50 total layers that hierarchically filter visual inputs. By utilizing an established framework proven successful at pattern detection from images, we inherit capabilities to extract generalizable features like colors, edges and shapes to recognize animal body parts. As the DeepLabCut developers highlight, ResNet-50 offers top performance on diverse behavioral tracking datasets (Mathis et al., 2018; Mathis, 2021). Compared to larger ResNets, its shallower depth prevents overfitting given limited training data while maintaining accuracy. For typical laboratory use cases, ResNet-50 struck the right balance between speed, memory efficiency, and precision in their validation. Thus we adopted the standard recommendation to use this network backbone for robust pose estimation.

We activated k-means clustering techniques alongside ResNet-50 training to select a diverse 380-frame subset from the hand-labeled data. Explicitly exposing models to challenging edge cases through smart sampling improves real-world performance. Our model then underwent 410,000 optimization rounds where training images pass through ResNet-50, body part coordinates get predicted, and labeling errors prompt incremental weight changes to minimize mistakes. This customized tuning process evolves the network from generic visual recognition to precise mouse limb identification. We allotted 410,000 iterations based on computing constraints, though the model’s labeling loss had sufficiently converged. We quantified optimization success using DeepLabCut’s “Evaluate Network” analysis on a held-out frame subset. This measured average Euclidean pixel distances between network-predicted coordinates and true hand-labels. Our reported training error of 1.43 pixels (px) and test error of 6.76 px indicates precise performance—predictions stayed closely aligned with ground truth annotations. By validating on excluded data, Evaluate Network confirms strong generalization prior to deploying models for analysis.

### Simple Behavioral Analysis

Following DLC body part identification, each of the 96 labeled recordings was analyzed with Simple Behavioral Analysis (SimBA, v1.3.0). SimBA processing commences by loading exported DeepLabCut CSV files containing (*x*, *y*) coordinate positions of labeled body parts across all video frames. This anatomical pose data feeds into a multi-stage machine learning workflow for detecting targeted behaviors.

Following standard guidelines recommended by SimBA’s developers (Nilsson et al., 2020), the first step trains ensemble models called Random Forests (RF). Ensembles combine predictions from many decision trees—flowcharts dividing frames into behavioral categories using body part positions. Our model instantiated 100 trees, defined by the “RF n estimators” parameter. Each tree determines optimal data splits differently: “auto RF max features” selects the number pose measures evaluated when identifying ideal separating points. The “gini impurity criterion” metric then judges proposed splits at tree nodes. We also provide 20% of sequences (“test size” parameter) for validating model generalizability, i.e., checking performance on entirely new unseen videos. Trees terminate when additional divisions no longer enhance separation: we required >1,000 frames per final group (“sample leaf parameter”).

With the RF model architecture configured, we create a specialized grooming detector. The network uses pose sequences manually-tagged as grooming to determine patterns distinguishing it from other behaviors. Optimized detection thresholds for grooming sequences (>0.75 confidence, >1,000 ms duration) are set using researcher evaluation of agreement with true grooming evidence. Once successfully trained, this tailored classifier automatically identifies and timestamps potential grooming bouts across all untagged videos. Outputted CSVs detail start frame, stop frame, duration and confidence score for all classified grooming occurrences—enabling downstream statistical analysis.

### HomeCageScan

The HomeCageScan (HCS) software enables simultaneous video recording and behavioral classification in real-time. Per correspondence with CleverSys technical staff, real-time quantification performs identically to traditional offline analysis (future validation can confirm this claim). As real-time analysis is the default configuration, we leveraged this mode for classifying grooming concurrently with video tracking during the 10 min recordings for each mouse.

HCS identifies complex behaviors like grooming based on frame-by-frame body part recognition and posture analysis relative to empirically tuned classification models. We used the default model parameters for detecting grooming and related behaviors out-of-the-box without modification. One key setting is the “groom cycle minimum size” lower bound threshold on the duration of conjoined frames grouped into a single bout. This was left at the default of 30 frames, equating to 1 s at 30 fps. Thus, any potential grooming episodes shorter than 1 s were not classified by the software.

Additionally, the “groom maintenance fraction” stipulates what percentage of frames within a bout must satisfy grooming pose criteria for the entire bout to be designated grooming. This was left at the default 80%, meaning grooming pose had to be maintained for at least 80% of frames within an episode lasting over 1 s in order to be categorized.

### Manual-scoring protocol

Behavioral Observation Research Interactive Software (BORIS, v7.13) was used to manually label each of the 96 videos. Two measurements of self-grooming were quantified: total number of grooming bouts and total duration spent grooming. Prior to observation, each video was assigned a randomly-generated name to blind the experimenter to the treatment condition of the subject, using an open-source Perl script (Salter, 2016). Each video was observed twice: once documenting the start and stop times of each grooming bout, and once documenting the start and stop times of each grooming bout as well as location of grooming. This produced the total number of overall grooming bouts/duration. For our analysis, we established that a grooming bout must last at least 1 s to be included in the total count and duration of grooming bouts.

### Statistics

To analyze grooming with each quantification method, we used a repeated measures ANOVA with a Greenhouse–Geisser correction; sex was the sole between-subjects factor. “Baseline” is defined as a 10 min observation of unmanipulated mice, directly preceding treatment manipulation (or “induction”). “Induction” refers to a 10 min observation directly following either treatment condition (restraint stress or water spray). “Treatment” condition refers to the specific stress treatment: restraint stress or water spray. Thus, the analysis included two within-subjects factors: treatment (water spray or restraint stress) and baseline/induction (baseline or induction). A *p*-value less than 0.05 was considered statistically significant.

Results from the 3 analysis methods (HCS, DLC/SimBA, and manual scoring) were compared using 2 one-sample *t*-tests (comparing each automated method to manual scoring) and a 3-way ANOVA followed by post-hoc *t*-tests. The 3-way ANOVA consisted of three factors: baseline or induction, treatment (restraint or water-spray), and analysis method (manual, HCS, or SimBA). Sex proved not to be a significant predictor of grooming in analyses within each analysis method and therefore was not included as a between-subject factor in this three-way analysis. Pearson correlations were calculated between measures. All analyses were conducted using IBM SPSS Statistics version 28 for statistical testing and Python 3.12.0 for data processing and visualization. In Python, we utilized the libraries NumPy for numerical computations, Scikit-Learn for linear regression, and Matplotlib for graph creation.

## Results

### Treatment effects vary depending on analysis method

First, the effects of the restraint and water-spray treatments were evaluated using manual scoring, HCS, and DLC/SimBA, independent of one another.

**Figure 1:**
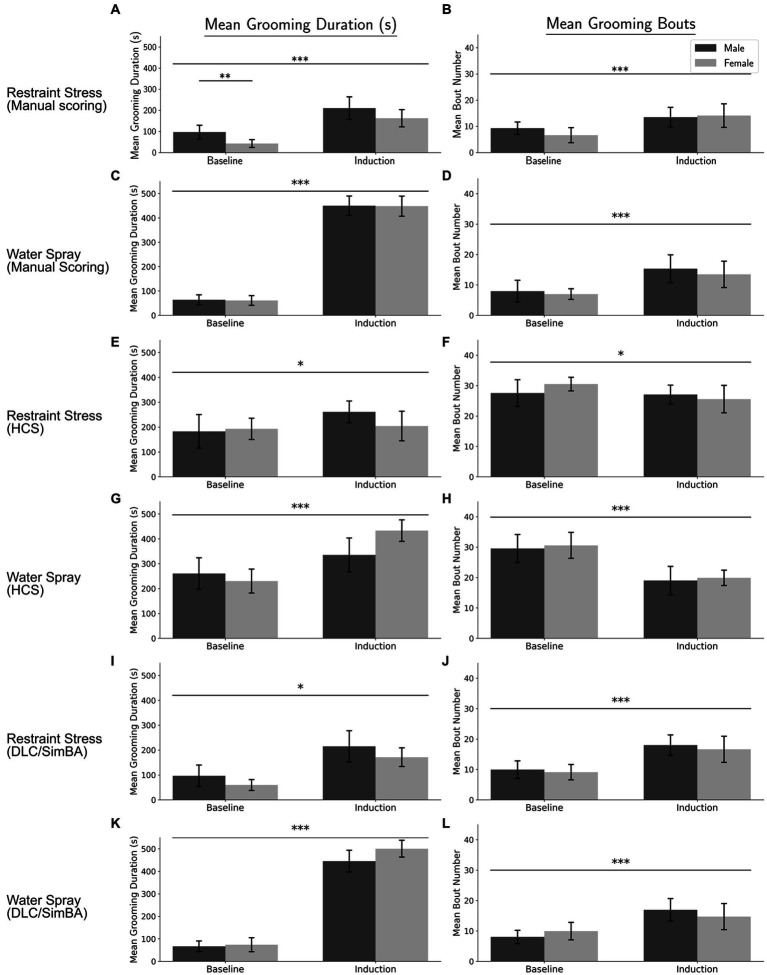
Grooming duration and bouts across conditions per analysis method. **(A)** Manual scoring quantification of grooming duration for the restraint condition. **(B)** Manual scoring quantification of grooming bouts for the restraint condition. **(C)** Manual scoring quantification of grooming duration for the water-spray condition. **(D)** Manual scoring quantification of grooming bouts for the water-spray condition. **(E)** HCS quantification of grooming duration for the restraint condition. **(F)** HCS quantification of grooming bouts for the restraint condition. **(G)** HCS quantification of grooming duration for the water-spray condition. **(H)** HCS quantification of grooming bouts for the water-spray condition. **(I)** DLC/SimBA quantification of grooming duration for the restraint condition. **(J)** DLC/SimBA quantification of grooming bouts for the restraint condition. **(K)** DLC/SimBA quantification of grooming duration for the water-spray condition. **(L)** DLC/SimBA quantification of grooming bouts for the water-spray condition. s = seconds. Dark Gray = Male, Light Gray = Female. Error bars are 95% confidence intervals. ^*^p < 0.05, ^**^p < 0.01, and ^***^p < 0.001. Intra-condition significance line (spans two bars) indicates post-hoc sex difference analysis. Inter-condition significance lines (spanning all four bars) indicates post-hoc main effect of condition (Baseline vs. Induction), for the specified treatment condition.

Mean grooming durations across conditions (two baselines, restraint, water-spray), scored manually, were analyzed by repeated measures ANOVA, with sex as a between-subject factor and treatment and baseline/induction as within-subject variables. There were main effects of both treatment and baseline/induction, as well as interaction effects of treatment × baseline/induction and treatment × sex (Figures 1A,C and Table 1). Both water-spray and restraint stress significantly increased grooming duration compared to baseline [*t*(70) = −31.396, *p* < 0.001, *d* = −7.888 and *t*(70) = −8.656, *p* < 0.001, *d* = −1.797 respectively]. Males groomed significantly more than females in the baseline condition [*t*(22) = 3.067, *p* < 0.01, *d* = 0.593]. Water-spray induced longer grooming durations than restraint stress [*t*(46) = −12.982, *p* < 0.001, *d* = −3.748]. A parallel analysis of grooming bouts (Figures 1B,D and Table 1) revealed a significant effect of baseline/induction, but not of sex or treatment. Both water-spray and restraint stress significantly increased grooming bouts from baseline [*t*(70) = −5.11, *p* < 0.001, *d* = −1.241 and *t*(70) = −4.838, *p* < 0.001, *d* = −1.086, respectively], but there was no significant difference between treatment conditions.

**Table 1:** Comparative analysis of grooming behavior measurement methods.

Figures	Dependent variable	Test used	Independent variables	*n*	*p*-value	*F* values
Figures 1B,D	Bouts (Manual Scoring)	Repeated Measures ANOVA	Treatment	24	0.953	*F*(1, 23) = 0.004
			Baseline/Induction		<0.001	*F*(1, 23) = 32.632
			Treatment × Baseline/Induction × Sex		Interaction: *p* = 0.346	*F*(1, 23) = 0.927
			Treatment × Baseline/Induction		Interaction: *p* = 0.601	*F*(1, 23) = 0.281
			Treatment × Sex		Interaction: *p* = 0.859	*F*(1, 23) = 0.032
			Baseline/Induction × Sex		Interaction: *p* = 0.620	*F*(1, 23) = 0.252
Figures 1F,H	Bouts (HCS)	Repeated Measures ANOVA	Treatment	24	0.21	*F*(1, 23) = 6.213
			Baseline/Induction		<0.001	*F*(1, 23) = 44.765
			Treatment × Baseline/Induction × Sex		Interaction: *p* = 0.543	*F*(1, 23) = 0.469
			Treatment × Baseline/Induction		Interaction: *p* = 0.011	*F*(1, 23) = 0.765
			Treatment × Sex		Interaction: *p* = 0.916	*F*(1, 23) = 0.011
			Baseline/Induction × Sex		Interaction: *p* = 0.257	*F*(1, 23) = 1.354
Figures 1J,L	Bouts (SimBA)	Repeated Measures ANOVA	Treatment	24	0.312	*F*(1, 23) = 1.070
			Baseline/Induction		<0.001	*F*(1, 23) = 32.225
			Treatment × Baseline/Induction × Sex		Interaction: *p* = 0.395	*F*(1, 23) = 0.751
			Treatment × Baseline/Induction		Interaction: *p* = 0.632	*F*(1, 23) = 0.236
			Treatment × Sex		Interaction: *p* = 0.916	*F*(1, 23) = 0.011
			Baseline/Induction × Sex		Interaction: *p* = 0.350	*F*(1, 23) = 0.910
Figures 1A,C	Duration (Manual Scoring)	Repeated Measures ANOVA	Treatment	24	<0.001	*F*(1, 23) = 131.264
			Baseline/Induction		<0.001	*F*(1, 23) = 577.628
			Treatment × Baseline/Induction × Sex		Interaction: *p* = 0.921	*F*(1, 23) = 0.010
			Treatment × Baseline/Induction		Interaction: *p* = <0.001	*F*(1, 23) = 143.801
			Treatment × Sex		Interaction: *p* = 0.043	*F*(1, 23) = 4.604
			Baseline/Induction × Sex		Interaction: *p* = 0.899	*F*(1, 23) = 0.016
Figures 1E,G	Duration (HCS)	Repeated Measures ANOVA	Treatment	24	0.011	*F*(1, 23) = 7.603
			Baseline/Induction		<0.001	*F*(1, 23) = 48.628
			Treatment × Baseline/Induction × Sex		Interaction: *p* = 0.012	*F*(1, 23) = 7.599
			Treatment × Baseline/Induction		Interaction: *p* = 0.005	*F*(1, 23) = 9.814
			Treatment × Sex		Interaction: *p* = 0.136	*F*(1, 23) = 2.396
			Baseline/Induction × Sex		Interaction: *p* = 0.154	*F*(1, 23) = 2.176
Figures 1I,K	Duration (SimBA)	Repeated Measures ANOVA	Treatment	24	<0.001	*F*(1, 23) = 167.965
			Baseline/Induction		<0.001	*F*(1, 23) = 387.368
			Treatment × Baseline/Induction × Sex		Interaction: *p* = 0.251	*F*(1, 23) = 1.389
			Treatment × Baseline/Induction		Interaction: *p* = <0.001	*F*(1, 23) = 162.588
			Treatment × Sex		Interaction: *p* = 0.003	*F*(1, 23) = 11.245
			Baseline/Induction × Sex		Interaction: *p* = 0.440	*F*(1, 23) = 0.618
Figure 4	Duration	Repeated Measures ANOVA	Treatment	24	<0.001	*F*(1, 23) = 78.543
			Analysis Method		<0.001	*F*(1, 23) = 79.770
			Baseline/Induction		<0.001	*F*(1, 23) = 460.957
			Treatment × Analysis Method × Baseline/Induction		Interaction: *p* < 0.001	*F*(1, 23) = 20.039
			Treatment × Analysis Method		Interaction: *p* < 0.001	*F*(1, 23) = 23.432
			Treatment × Baseline/Induction		Interaction: *p* < 0.001	*F*(1, 23) = 106.700
			Analysis Method × Baseline/Induction		Interaction: *p* < 0.001	*F*(1, 23) = 103.869
Figure 7	Bouts	Repeated Measures ANOVA	Treatment	24	0.102	*F*(1, 23) = 2.899
			Analysis Method		<0.001	*F*(1, 23) = 187.255
			Baseline/Induction		0.006	*F*(1, 23) = 9.337
			Treatment × Analysis Method × Baseline/Induction		Interaction: *p* = 0.004	*F*(1, 23) = 8.806
			Treatment × Analysis Method		Interaction: *p* = 0.080	*F*(1, 23) = 2.905
			Treatment × Baseline/Induction		Interaction: *p* = 0.190	*F*(1, 23) = 1.827
			Analysis Method × Baseline/Induction		Interaction: *p* < 0.001	*F*(1, 23) = 57.577

This table presents a detailed comparison of the outcomes from different grooming behavior measurement methods, including manual scoring, HomeCageScan (HCS), and DeepLabCut/Simple Behavioral Analysis (DLC/SimBA). The table highlights differences in measuring grooming duration and bouts under various treatment conditions.

For grooming duration as quantified using HCS, there were significant main effects of treatment condition and baseline/induction, a two-way interaction effect of treatment × baseline/induction, and a three-way interaction effect of treatment × baseline/induction × sex (Figures 1E,G and Table 1). Post-hoc pairwise comparisons revealed significant differences between conditions: water-spray > baseline, *t*(70) = −6.317, *p* < 0.001, (*d* = −1.57); restraint > baseline, *t*(70) = −2.006, *p* < 0.05, (*d* = −0.502); water-spray > restraint, *t*(46) = −3.948, *p* < 0.001, (*d* = 1.140). These results are qualitatively similar to those found with manual scoring, though the between-condition effect sizes are smaller.

HCS diverged from manual scoring in its findings for grooming bouts (Figures 1F,H and Table 1). Total grooming bouts differed not only between baseline/induction, as in manual scoring, but also between restraint and water-spray treatments. There were no significant effects of sex according to HCS. Like manual scoring, HCS identified significant differences between baseline and water-spray bouts, *t*(70) = 6.685, *p* < 0.001 (*d* = 1.677), and baseline and restraint bouts, *t*(70) = 2.14, *p* < 0.05 (*d* = 0.476). However, HCS reports that both water-spray and restraint resulted in *decreased* grooming bouts, whereas manual scoring suggests that water-spray and restraint resulted in *increased* grooming bouts. Furthermore, HCS found an additional significant difference between restraint and water-spray bouts, *t*(46) = 3.939, *p* < 0.001 (*d* = −1.137), with water-spray inducing a significantly fewer number of bouts; this is in contrast to manual scoring, which did not identify a significant bout difference between treatments.

Grooming duration quantified using DLC/SimBA differed across treatment and baseline/induction conditions (Figures 1I,K and Table 1). Like manual scoring and HCS, DLC/SimBA identified significant pairwise differences in grooming duration: water-spray > baseline, *t*(70) = −15.749, *p* < 0.001; restraint > baseline, *t*(70) = −15.749 (*d* = −1.619), *p* < 0.05; water-spray > restraint, *t*(46) = −12.367 (*d* = −3.570), *p* < 0.001 (*d* = −6.677). Total grooming bouts as measured using DLC/SimBA also differed between baseline/induction conditions (Figures 1J,L and Table 1). Like manual scoring, and unlike HCS, DLC/SimBA found that there was not a significant difference in bouts between treatment conditions, *t*(46) = 0.0849, *p* = 0.4. Also like manual scoring, DLC/SimBA found that water-spray significantly increased the number of grooming bouts compared to baseline, *t*(70) = −2.496, *p* < 0.05, *d* = −1.282; furthermore, DLC/SimBA did not identify a significant difference between restraint induction and water-spray induction, *t*(70) = −1.503, *p* = 0.137.

Thus, neither HCS and DLC/SimBA fully recapitulated the results of manual scoring: manual scoring found a significant increase in bouts in water-spray, HCS found a significant decrease in bouts in water-spray, and neither HCS nor DLC/Simba identified the sex difference in grooming duration identified by manual scoring.

### DLC/SimBA more accurately predicts total grooming duration than HCS.

**Figure 2:**
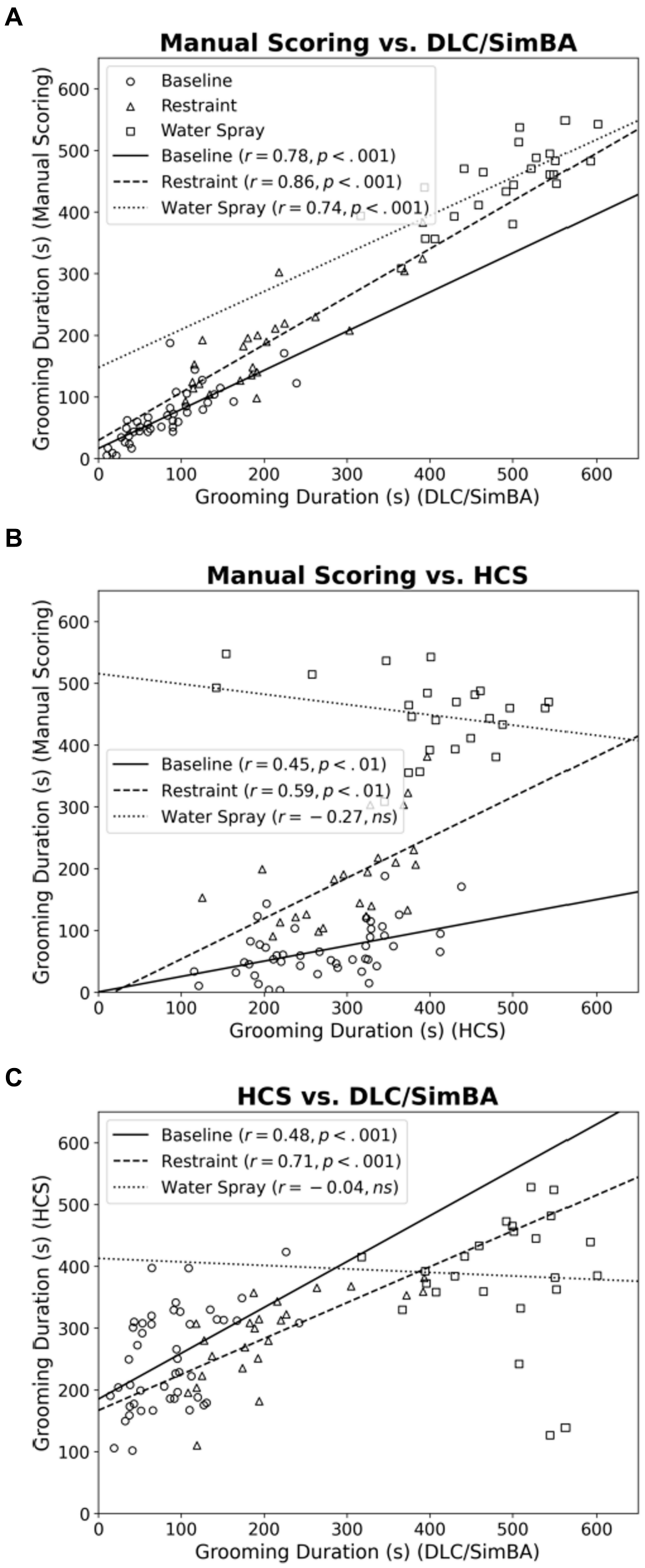
Correlations between methods of quantifying grooming duration, categorized by treatment group. **(A)** Manual Scoring vs. DLC/SimBA. **(B)** Manual Scoring vs. HCS. **(C)** HCS and DLC/SimBA. s, seconds. Data points: Circle = Baseline, Triangle = Restraint, Square = Water Spray. Regression Lines: Solid = Baseline, Dashed = Restraint, Dotted = Water Spray.

Across all treatment conditions, manual scoring and DLC/SimBA measurements of grooming duration were strongly positively correlated (Figure 2A; baseline, *r*(46) = 0.781, *p* < 0.001; restraint, *r*(22) = 0.857, *p* < 0.001; water spray, *r*(22) = 0.744, *p* < 0.001). This suggests that DLC/SimBA closely aligns with manual scoring in calculating total grooming duration.

For the correlation between measurements of manual and HCS grooming duration, only baseline and restraint stress conditions were significant (Figure 2B; baseline, *r*(46) = 0.449, *p* = 0.002; restraint, *r*(22) = 0.589, *p* = 0.004; water spray, *r*(22) = −0.274, *p* = 0.195). This is consistent with our past results (Xu et al., 2015). Notably, HCS consistently overscored grooming in the baseline condition, when grooming levels are low, but consistently underscored grooming in the water-spray condition, when grooming levels are high.

HCS and DLC/SimBA measures of grooming duration were also only correlated in the baseline and restraint stress conditions, but not for the water spray condition (Figure 2C; baseline, *r*(46) = 0.480, *p* < 0.001; restraint, *r*(22) = 0.710, *p* < 0.001; water spray, *r*(22) = −0.042, *p* = 0.852).

### HCS and DLC/SimBA vary in their treatments of low and high grooming durations.

**Figure 3:**
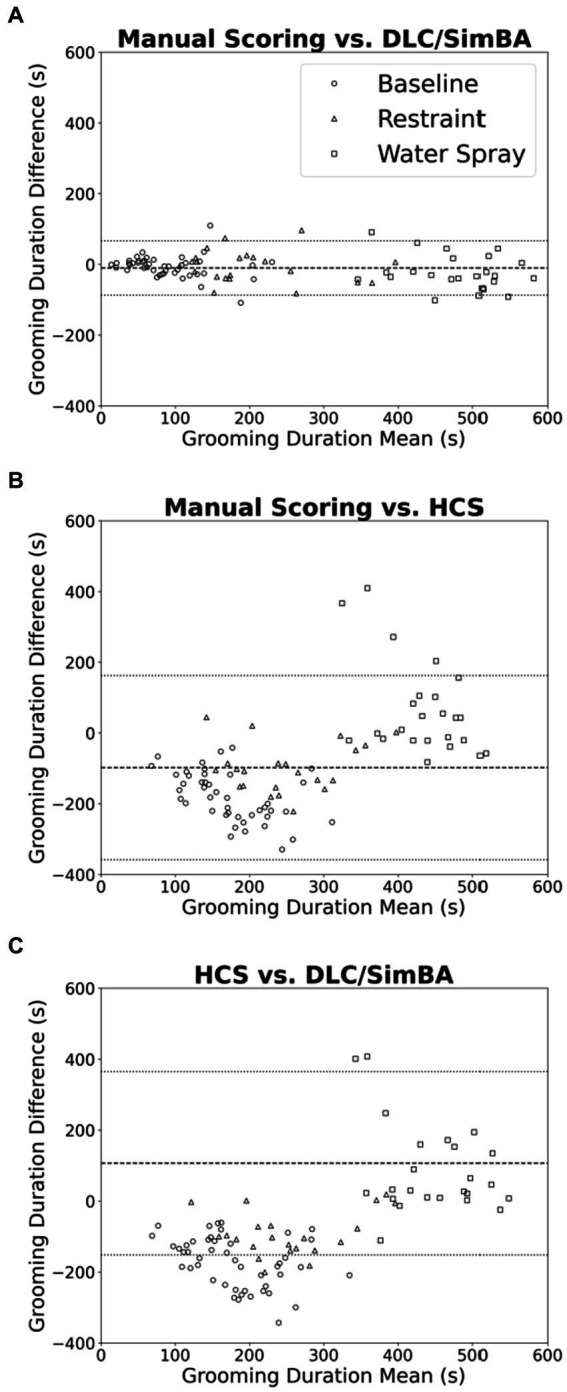
Bland–Altman plots comparing analysis methods (grooming duration). **(A)** Manual Scoring vs. DLC/SimBA. **(B)** Manual Scoring vs. HCS. **(C)** HCS vs. DLC/SimBA. Dashed line indicates mean difference and dotted lines indicate 95% confidence intervals. s, seconds. Circle = Baseline, Triangle = Restraint, Square = Water Spray.

We used Bland–Altman plots to further characterize relationships between scoring methods when quantifying grooming duration. When comparing manual scoring vs. DLC/SimBA (Figure 3A), there is only a weak relationship of points above and below the mean, at both high and low grooming levels [*r*(94) = −0.235, *p* = 0.021].This suggests that DLC/SimBA exhibits a slight bias towards under-estimating grooming duration at high grooming levels, relative to manual scoring. The mean difference between the duration of grooming calculated by manual scoring and by DLC/SimBA was significant, with a mean difference of −10.23 s [*t*(95) = 11.392, *p* < 0.001], suggesting that DLC/SimBA tends to slightly overestimate grooming durations relative to manual scoring.

Figure 3B presents the Bland–Altman plot for manual scoring and HCS. There was a positive correlation between average and difference scores [*r*(94) = 0.562, *p* < 0.001], suggesting that HCS overestimates at low grooming durations and underestimates at high grooming durations. The mean difference between grooming durations calculated by manual scoring and HCS was −98.50 s, suggesting HCS dramatically overestimates grooming durations overall. Finally, the Bland–Altman plot comparing HCS to DLC/SimBA (Figure 3C) follows a negative slope [*r*(94) = 0.616, *p* < 0.001]. This indicates that at low grooming durations, HCS reports greater grooming duration than SimBA, and at higher grooming durations, DLC/SimBA reports greater grooming durations than HCS. The mean difference in duration calculated by HCS and DLC/SimBA was 107.40 s, further underscoring the divergence of these methods from one another.

### HCS and DLC/SimBA vary in their estimation of grooming duration at baseline but not during provoked grooming

**Figure 4:**
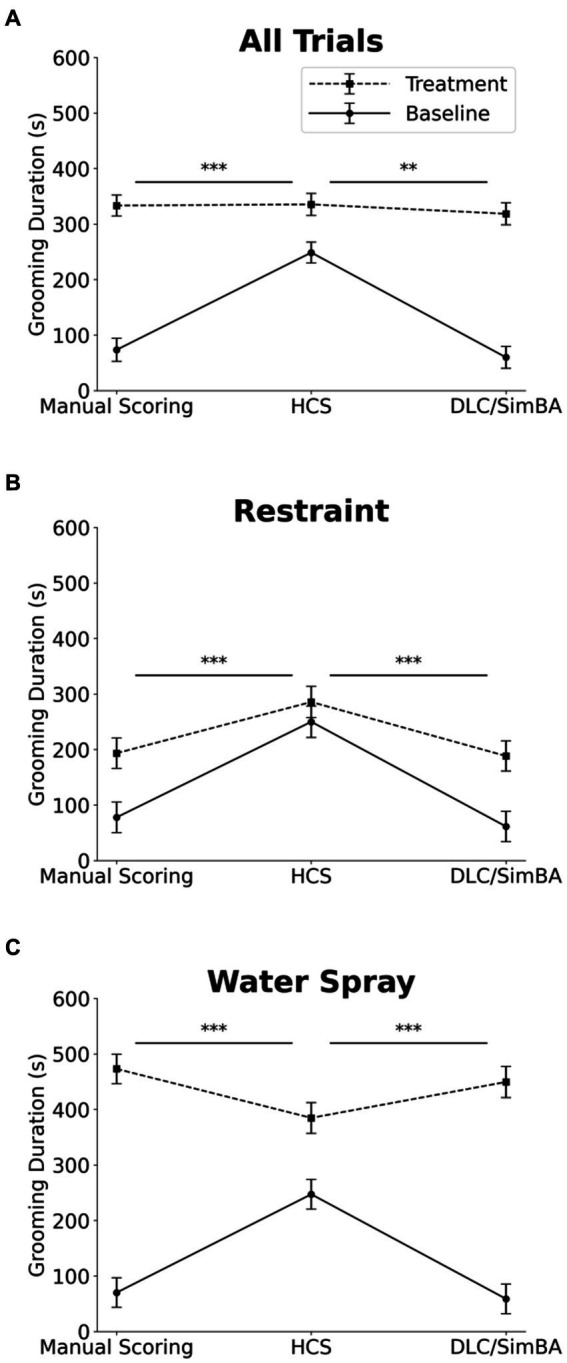
Impact of analysis methods on interpreting treatment-induced effects on grooming duration. Comparison of analysis methods to assess grooming duration across treatment conditions **(A)** All trials. **(B)** Restraint trials only. **(C)** Water-spray trials only. s, seconds. Solid Line = Baseline, Dashed Line = Treatment. ^*^p < 0.05, ^**^p < 0.01, and ^***^p < 0.001. Error bars: 95% confidence interval.

We next analyzed grooming measures across all conditions. Three-way within-subject ANOVA (restraint vs. water-spray, baseline vs. induced, HCS vs. DLC/SimBA vs. manual scoring), revealed a significant two-way interaction between analysis method and grooming induction: HCS reported significantly higher grooming durations at baseline, relative to DLC/SimBA and manual scoring, but did not differ as dramatically after grooming induction (Figure 4A and Table 1). This pattern was also apparent when restraint-induced and spray-induced grooming were examined separately (Figures 4B,C and Table 1). HCS tended to report *higher* mean grooming duration than manual scoring/SimBA in restraint trials and a *lower* mean grooming duration than manual scoring/SimBA in water-spray trials, suggesting that HCS may truncate the range of grooming durations.

### SimBA more accurately predicts total grooming bouts than HCS

**Figure 5:**
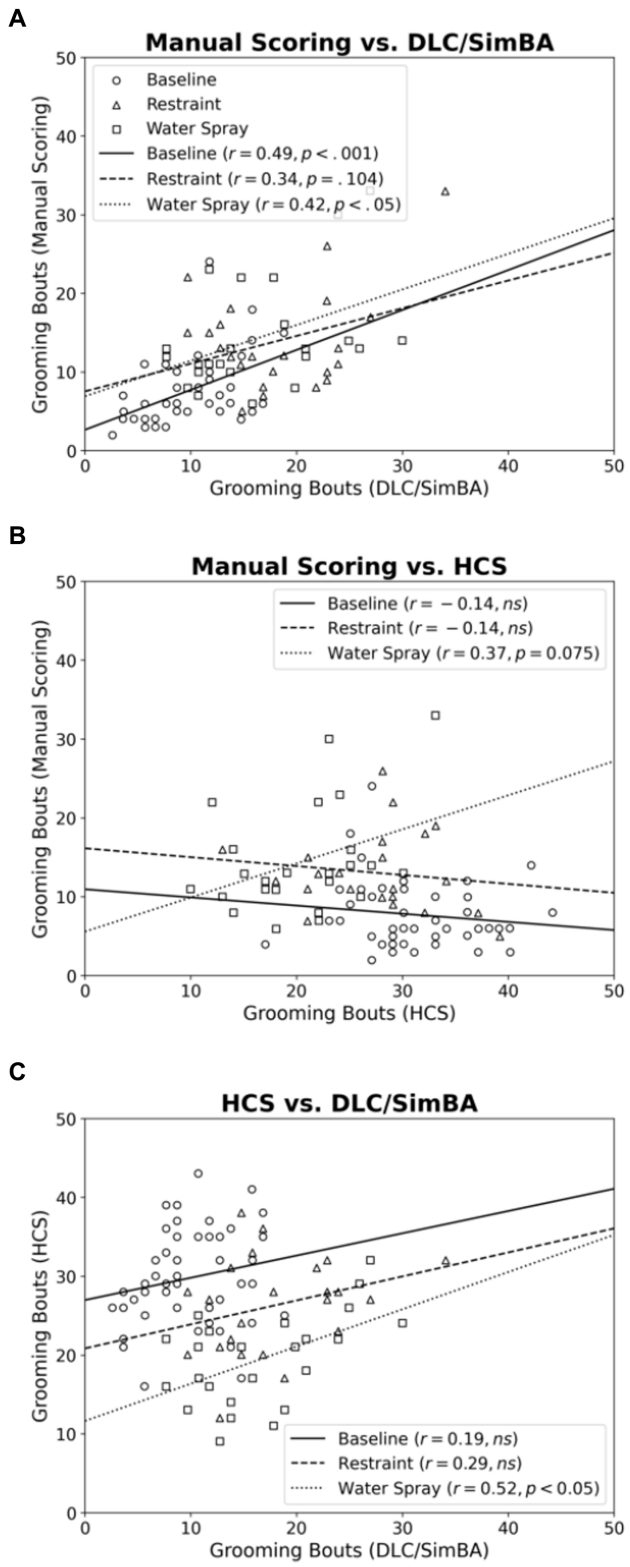
Correlations between methods of quantifying grooming bouts, categorized by treatment group. **(A)** Manual Scoring vs. DLC/SimBA. **(B)** Manual Scoring vs. HCS. **(C)** HCS and DLC/SimBA. Data Points: Circle = Baseline, Triangle = Restraint, Square = Water Spray. Regression Lines: Solid = Baseline, Dashed = Restraint, Dotted = Water Spray.

Next we compared the scoring methods in their assessment of grooming bouts. Across baseline and water spray conditions, manual scoring and DLC/SimBA measures of bout number were positively correlated [Figure 5A; baseline, *r*(46) = 0.487, *p* < 0.001; water spray, *r*(22) = 0.423, *p* = 0.049]. This correlation reached trend levels for the restraint stress condition [*r*(22) = 0.340, *p* = 0.104].

For baseline and restraint stress conditions, there was no significant correlation between manual scoring and HCS measures of grooming bouts [Figure 5B; baseline, *r*(46) = −0.137, *p* = 0.187; restraint, *r*(22) = −0.142, *p* = 0.528]. However, there was a trend-level positive correlation in the water spray condition [*r*(22) = 0.371, *p* = 0.075].

For baseline and restraint stress conditions, there was no correlation between SimBA and HCS scores of grooming bouts, however there was a significant association for the water spray condition [Figure 5C; baseline, *r*(46) = 0.192, *p* = 0.201; restraint, *r*(22) = 0.292, *p* = 0.187; water spray, *r*(22) = 0.519, *p* = 0.013].

### HCS and DLC/SimBA vary in their treatments of low and high grooming bouts

**Figure 6:**
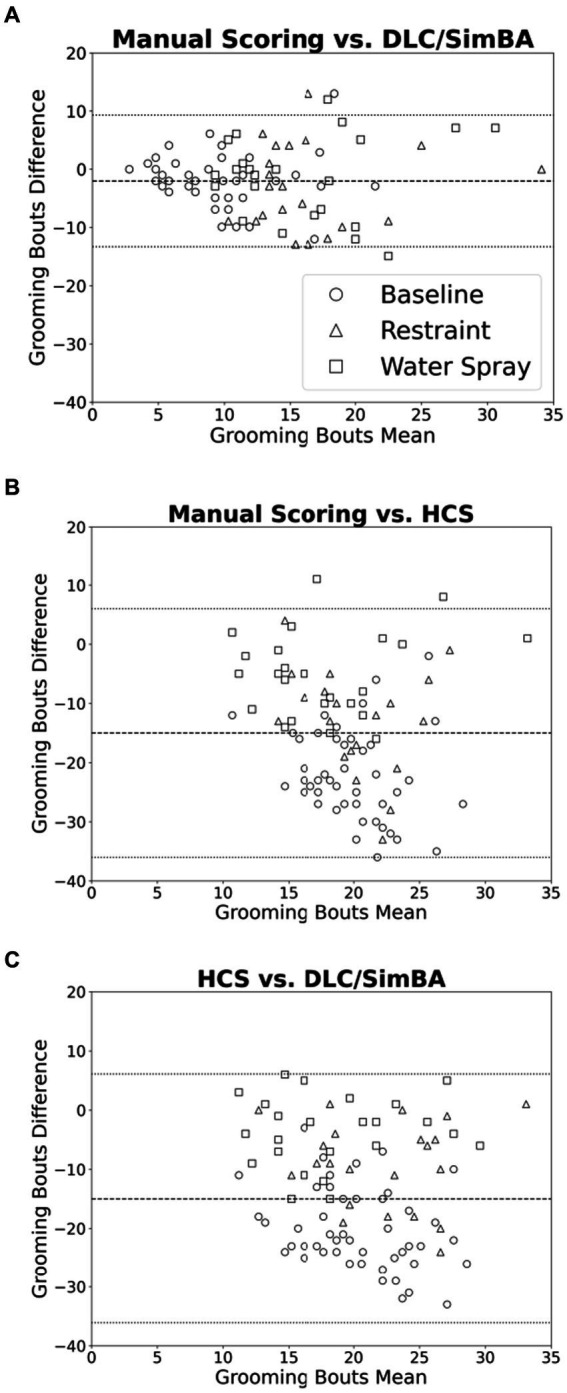
Bland–Altman plots comparing analyses methods (grooming bouts). **(A)** Manual Scoring vs. DLC/SimBA. **(B)** Manual Scoring vs. HCS. **(C)** HCS vs. DLC/SimBA. Dashed line indicates mean difference and dotted lines indicate 95% confidence intervals. Circle = Baseline, Triangle = Restraint, Square = Water Spray.

Bland–Altman plots were used to further visualize differences in measured grooming bouts across scoring methodologies. Comparing manual scoring to DLC/SimBA (Figure 6A), there was a roughly even distribution of data points with no overall trend in overestimation or underestimation. However, there is a cluster of points in the upper right of the plot, suggesting that at high levels of grooming, DLC/SimBA may underestimate bout number. The mean difference between number of bouts calculated by manual scoring and SimBA was significant [mean difference = −1.98; *t*(95) = 16.9, *p* < 0.001], suggesting that, overall, SimBA slightly overestimates bout number. In comparing manual scoring to HCS, the data points again appear evenly distributed. However, the mean difference in the number of bouts between manual scoring and HCS was −15.3 [Figure 6B; *t*(95) = 35.2, *p* < 0.001], indicating that HCS substantially overestimates grooming bouts overall. The mean difference in grooming duration between HCS and DLC/SimBA was 13.3 [Figure 6C; *t*(95) = 28.9, *p* < 0.001], suggesting that on average HCS tends to score grooming bouts higher than DLC/SimBA.

### HCS overestimates grooming bouts in baseline conditions

**Figure 7:**
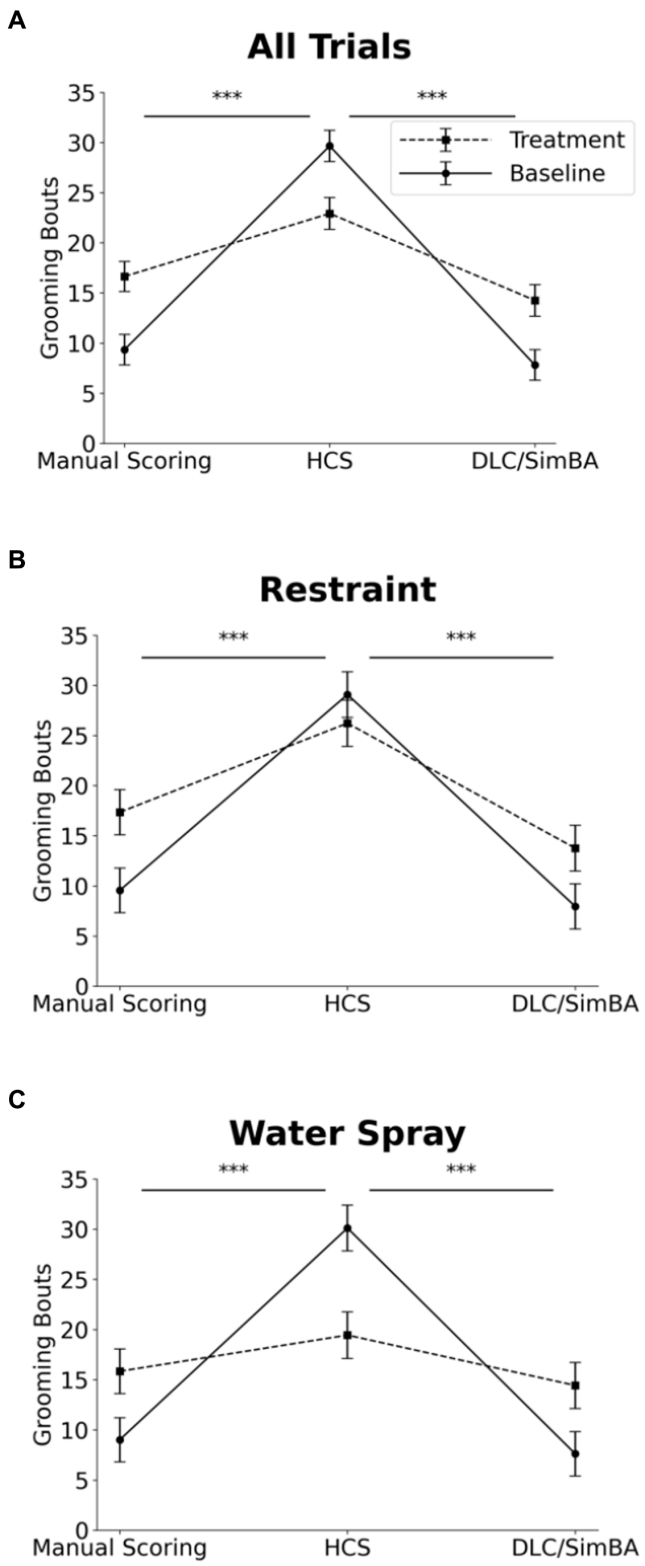
Impact of analysis methods on interpreting treatment-induced effects on grooming bouts. Comparison of analysis methods to assess grooming bouts across treatment conditions. **(A)** All trials. **(B)** Restraint trials only. **(C)** Water-spray trials only. Solid Line = Baseline, Dashed Line = Treatment. ^*^p < 0.05, ^**^p < 0.01, and ^***^p < 0.001. Error bars: 95% confidence interval.

Three-way within subjects ANOVA (restraint vs. water-spray, baseline vs. induced, HCS vs. DLC/SimBA vs. manual scoring) on grooming bouts revealed a significant two-way interaction between analysis method and baseline/induced: HCS dramatically overreported grooming bouts at baseline, relative to the other methodologies, but not after induction—indeed, HCS reported more grooming bouts at baseline than after restraint or water-spray (Figure 7 and Table 1; see also Figure 1H).

## Discussion

Abnormal repetitive grooming behaviors in mouse models of disease pathophysiology have been interpreted as paralleling abnormal repetitive behaviors seen in human psychiatric disorders. The increasing importance of such behaviors underscores the need for robust, automated behavioral quantification techniques. In this paper, we compared the assessment of mouse self-grooming by an open-source method [DeepLabCut(DLC)/SimBA] and a commercial method [HomeCageScan (HCS)], compared to the current standard of manual scoring by a blinded rater.

All three methods confirmed that both restraint and water-spray conditions elevated grooming duration compared to baseline, with water-spray inducing more grooming than restraint; this is in alignment with current literature that suggests physical stressors induce more grooming behavior than emotional stressors (Mu et al., 2020). Previous work has documented 4 mists as sufficient to induce elevated grooming (van Erp et al., 1994), while we chose to use 30 mists in order to more confidently induce increased grooming. The three methods disagreed on changes in grooming bout count. Manual scoring and DLC/SimBA showed that both treatment conditions resulted in an increase in grooming instances from baseline, whereas HCS suggested a decrease in grooming bouts from baseline.

While DLC/SimBA largely aligned with manual scoring in terms of grooming duration and the number of grooming bouts, notable discrepancies were observed between HCS and manual scoring. Specifically, HCS showed a significant relationship with manual estimates of the number of grooming bouts in the water spray condition, but this did not extend to the duration of grooming. Conversely, HCS was significantly in sync with manual duration estimates during baseline and restraint stress conditions, yet it diverged from manual counts of grooming bouts in these scenarios. This discrepancy may stem from the heightened responsiveness of DLC/SimBA compared to HCS to the nuanced differences in grooming microstructure patterns that manifest following water spray as opposed to restraint stress (Mu et al., 2020). Future studies should explore if DLC/SimBA can detect specific elements of the grooming sequence with greater acuity than HCS. Factors such as minimum bout duration settings and continuity thresholds may critically impact bout designations between approaches. The accuracy of bout identification is crucial for behavioral inferences, and understanding the sources of disagreement by independently and openly manipulating key parameters is an important avenue for future research. A possible explanation for these discrepancies is that HCS may struggle to accurately determine the end of a grooming duration, potentially combining multiple grooming events into a single bout. This could explain the reduced number of grooming bouts but increased duration observed. Additionally, when the animal’s back is turned to the camera, SimBA might mislabel breaks in grooming that resemble grooming postures (e.g., sniffing or crouching), leading to an artificial decrease in grooming bout number.

Furthermore, HCS’s minimum 1 s duration threshold for merging shorter bouts, along with stricter criteria for breakpoints compared to human judgment, might contribute to the observed differences in bout lengths and counts between manual scoring and SimBA. However, bout distinctions are influenced by multiple configuration factors across programs. Therefore, we should be cautious in making definitive statements about the sources of disagreement without specifically manipulating segmentation parameters, a task that is challenging with proprietary tools. This nuanced understanding of the limitations and variances in automated and manual scoring methods underscores the need for careful consideration in behavioral analysis.

The difficulty of automated analysis of self-grooming behavior in this study may be partially accounted for by the single side-view angle of the camera used in the experimental setup. Capturing behaviors from a side-facing angle is necessary for smaller, more intricate behaviors like grooming, which may be difficult to decipher from a top-down view. Yet, side-angle camera views present notable challenges to machine learning algorithms as body parts change size, disappear, and reappear into view as the animal moves towards and away from the camera. SimBA notes these difficulties, and their pre-packaged side-view body label set is not validated (Sturman et al., 2020). Similarly, HCS is advertised as the only automated side-angle behavioral analysis tool for mice on the market, reflecting the unique obstacles that a side-angle camera raises. The discrepancies introduced by the one side-angle camera setup may be a topic for further exploration, such as in comparison to recordings from a top-down view and from multiple side-angle cameras.

In general, the shift towards open-source software in behavioral analysis is a positive one. While commercial products act as a “black box” in which an experimenter can view the inputs and outputs but nothing in between, open-source tools allow for greater insight into analysis methods. In the case of DLC/SimBA, these programs allow for greater flexibility in algorithmic variables, such as the number of body-part labels to use, the type of neural network architecture to base the model on, and the final statistics to calculate (e.g., bout number and grooming duration), among others. Furthermore, when investigating unexpected results, open-source tools like DLC/SimBA allow users to look at individual steps of the program to determine at what point error was introduced—for example, with DLC/SimBA, we are able to examine the accuracy of body-part labeling by DLC as a checkpoint prior to using SimBA. In contrast, with HCS, it is unclear at what step aberrant results were introduced—whether this was at body part labeling, behavior labeling, or some point in between.

In general, open-source tools present a steeper learning curve to use, as the technical knowledge required to use them (e.g., how to use the terminal and open-source repositories like GitHub) presents additional challenges to neuroscientists. The SimBA README document is well designed and easy to follow, but it assumes a significant level of background knowledge to be able to get started. In this aspect, traditional commercial tools with exhaustive manuals may be more user-friendly to neuroscientists.

While the present investigation centered on the emerging open-source analysis solutions DeepLabCut and SimBA, expanding direct comparison to additional open source packages like VAME (Luxem et al., 2022), B-SOiD (Hsu and Yttri, 2021), and MoSeq (Wiltschko et al., 2020), which use varied supervised and unsupervised learning approaches to automated behavioral analysis, merits future work. Each employs distinct approaches to modeling body posture and classifying behavior that could complement or outperform SimBA. We selected SimBA specifically due to its widespread adoption evidenced by top-ranking search results and high citation count for “open source behavioral analysis” on both Google and Google Scholar. Contrasting how these alternatives generalize to novel housing situations relative to SimBA poses an important next step for benchmarking capability across tools developed in academic labs. Still, even commercial solutions like EthoVision (Noldus et al., 2001) warrant inclusion to evaluate performance among diverse proprietary and cost-free offerings. Systematically applying rigorous validation tests across leading techniques, open and closed alike, should yield an expanded, more articulate understanding of optimal solutions tailored to nuanced experimental requirements while pushing the boundaries of accessible automation for reproducible science.

In our analysis, the simplified cage conditions lacked enrichment elements that could challenge tracking. Comparing performance in complex environments with tunnels, nesting or running wheels poses an important direction as naturalistic stimuli further tax algorithms. Similarly, restricting recordings to 10 min sessions risks inadequately sampling complete behavioral repertoires expressed over longer periods. Continuous 24 h monitoring could provide more representative activity data for classifier training and testing. Finally, our sole focus on a single repetitive grooming behavior prohibits inferring flexible quantification of broad ethograms. Expanding analysis to social behaviors, anxiety-like activities and more would enhance understanding of commercial and open-source technique capabilities measuring comprehensive phenotypes. Further work incorporating enriched testing environments, prolonged observation and diverse behavioral categories will elevate applicability to wider translational questions.

When considering the current shift towards automated complex data analysis, it is also necessary to address the issues of scale associated with machine learning analyses. Processing the 96 10 min long videos in DeepLabCut resulted in the creation of over 50,000 files; the creation and long-term storage of these files pose a significant and costly technical challenge.

In sum, we find the combination of DLC with SimBA to provide an excellent parallel to manual scoring by a blinded observer when quantifying grooming duration in singly-housed mice, using a single side-view camera. DLC/SimBA is a potentially powerful tool for high throughput, rigorous analysis of grooming behavior in the context of pathophysiological and ethological studies. The measurement of grooming bouts was also positively correlated with manual scoring, though less strongly than that of grooming duration. As the field advances, there is a growing consensus on the best tools for behavior analysis, with the development of these and other analytic methods paving the way for more standardized, quantitative approaches in the study of disease pathophysiology.

## Data availability statement

The raw data supporting the conclusions of this article will be made available by the authors, without undue reservation.

## Ethics statement

The animal study was approved by Yale University Institutional Animal Care & Use Committee (IACUC). The study was conducted in accordance with the local legislation and institutional requirements.

## Author contributions

KC: Data curation, Investigation, Software, Writing – original draft. RW: Data curation, Writing – review & editing. CP: Funding acquisition, Supervision, Writing – review & editing. CF: Conceptualization, Funding acquisition, Supervision, Writing – review & editing.
